# Prevalence, Risk Factors and Microbiological Profile of Orthopaedic Surgical Site Infection in North-Eastern Peninsular Malaysia

**DOI:** 10.5704/MOJ.2211.015

**Published:** 2022-11

**Authors:** WC Chua, SA Rahman, ZZ Deris

**Affiliations:** 1 Department of Medical Microbiology and Parasitology, Universiti Sains Malaysia, Kubang Kerian, Malaysia; 2 Department of Orthopaedics, Universiti Sains Malaysia, Kubang Kerian, Malaysia

**Keywords:** orthopaedics surgical site infections, risk factors, prevalence, causative organisms

## Abstract

**Introduction:**

The devastating outcome of orthopaedic surgical site infections (SSI) are largely preventable if its risk factors, causative organisms and antimicrobial susceptibility patterns in the regional area are known.

**Materials and methods:**

We conducted a retrospective study to address the lack of epidemiological and microbiological data on orthopaedic SSI in Malaysia. All the 80 patients diagnosed and treated for microbiologically proven orthopaedic SSIs in a tertiary hospital in Malaysia from April 2015 to March 2019 were included in a 1:2 case control study.

**Results:**

The prevalence of SSI in clean and clean-contaminated surgeries was 1.243%, which is consistent with most of the studies worldwide, but is low compared to other studies done in Malaysia. The most common type of orthopaedics SSI were internal fixation infections (46.25%), superficial SSIs (25.2%) and Prosthetic joint infections (18.75%). Obesity and tobacco use were found to be significant risk factors of orthopaedic SSI. The most common perioperative prophylaxis used was IV cefuroxime. Majority of the cases (86.5%) received prolonged prophylactic antibiotics. The most common causative agent was Staphylococcus aureus (31.25%), followed by Pseudomonas aeruginosa (26.25%) and Enterobacter spp (7.5%). Methicillin-resistant Staphylococcus aureus (MRSA) accounted for 20% of the S. aureus infections. Up to 19.4% of the Gram-negative organisms are multidrug resistant. The higher rate of isolation of organisms resistant to the prophylactic antibiotics being used may be related to the prolonged use of prophylactic antibiotics, which exerted selective pressure for the acquisition of resistant organisms.

**Conclusion:**

Despite its relatively low prevalence in our local institution and worldwide, the prevention of SSI in orthopaedic practice is crucial to avoid morbidity, mortality and high healthcare cost. This may be achieved by control of modifiable risk factors such as obesity and tobacco use, appropriate use of prophylactic antibiotics and implementation of good surgical and infection control practices.

## Introduction

Surgical site infections (SSI) in orthopaedic practice, is a costly problem and is associated with significant morbidity, reduction in quality of life and often results in devastating outcome. It contributes to considerable morbidity and healthcare expense^1^. An additional 6.5 to 11.2 days of length of stay and cost of USD 20,785 per event are attributed to SSI, and the total attributable cost in the US in 2008 was USD 3.2 billion^2^.

The operative procedures can be stratified into clean, clean-contaminated, contaminated, and dirty to predict the bacterial load introduced into the operative site. Surgical site infections in orthopaedic practice may present with wound infection, which can be further categorised by CDC’s National Healthcare Safety Network into superficial and deep incisional wound infections^3^. However, infections unique to orthopaedic SSI is the infection of orthopaedics devices used for fracture stabilisation, vertebral fusion, joint replacement, and correction of deformities^4^. These implant related infections include periprosthetic joint infections and internal fixation associated infections.

The SSI is largely preventable if some of the information in the local settings such as risk factors, common causative organisms and their susceptibility patterns are known. Among modifiable risk factors that has been published in previous reports are pre-operative anaemia^5,6^, obesity^5,7-9^, renal disease^5^, diabetes^5,10,11^, tobacco use^10,12^, long operative time^13^ and coronary artery disease^10^.

This study examines local prevalence and risk factors associated with SSI in orthopaedic practice, thus more attention and resources can be focused on effective reduction of risk and prevention of the infection. This study also identified common pathogens and their sensitivity patterns for a better selection of empirical therapy.

## Materials and Methods

The approval of local institutional ethical committee was obtained for this study. The location of this study was an 800-bedded tertiary care hospital serving the population of north-eastern state of Peninsular Malaysia. The study population consist of all patients aged more than 12 years old who had undergone clean and clean-contaminated procedure in the hospital with microbiologically confirmed orthopaedic surgical site infection during the period of April 2015 to March 2019. All intra-operative specimens were sent to the hospital clinical microbiology laboratory. Intra-operative clinical specimens included were pus aspirate, tissue, synovial fluids, and bone specimens sent for culture and antibiotic susceptibility testing. Patients with prior orthopaedics skin and soft tissue infections, surgical site infections diagnosed by clinical or radiological criteria and those who underwent the index operative procedure in another centre were excluded.

The pus aspirate, tissue, synovial fluid, and bone specimens obtained intra-operatively during orthopaedic procedure were processed using standard microbiology techniques and inoculated into 5% sheep blood agar, MacConkey agar and incubated in 37°C in ambient air for 24 hours. A chocolate blood agar was inoculated and incubated in 37°C in 5-10% carbon dioxide for 24 hours. A separate sheep blood agar was inoculated and incubated in anaerobic condition for 48 hours. The causative organisms were identified using standard biochemical identification methods or automated identification system, Vitek214. The causative organisms isolated were tested for antimicrobial susceptibility as recommended by Clinical and Laboratory Standards Institute (CLSI) M100 document using Kirby-Bauer disc diffusion and gradient diffusion E-test [BioMérieux, Marcy-l'Étoile] methods^15^.

For the risk factors, a case-control study was performed. Patients with positive culture of the intra-operative clinical specimen who have been treated for orthopaedic surgical site infection were identified as case. The controls for a 1:2 case control study was chosen randomly among patients who had undergone orthopaedic surgery with culture negative intra-operative specimens and were matched by period of operative procedure within the same month.

Medical records of the cases and control were reviewed for the possibility of SSI and the presence or absence of risk factors. Surgical site infection (SSI) is defined as an infection related to an operative procedure that occurs at or near the surgical incision (incisional or organ/space) within 30 days of the procedure or within 90 days if prosthetic material is implanted at surgery^16^. Clean procedures are procedures in which no inflammation is encountered, and the respiratory, alimentary, genital, or uninfected urinary tract is not entered, and the wounds are primarily closed. Clean contaminated procedures are operations in which the respiratory, alimentary, genital, or urinary tracts are entered under controlled conditions and without unusual contamination, with no evidence of infection and major breaks in technique. The wounds should not be accidental or open.

Infectious Disease Society of America (IDSA) defines periprosthetic joint infection (PJI) as patients who underwent joint replacement procedures with presence of (1) a sinus tract communicating with the prosthetic joint, (2) purulence without another known aetiology surrounding the prosthetic device, (3) acute inflammation consistent with infection at histopathologic examination of periprosthetic tissue, (4) an elevated leukocyte count in synovial fluid or predominance of neutrophils in the synovial fluid, (5) growth of identical microorganism in at least two intra-operative cultures in case of a low-virulence microorganism, or in case of a virulent organisms, growth in a single specimen^17^. There is currently no clear definition for internal fixation associated infection. However, by analogy with PJI, Zimmerli *et al* proposed that internal fixation associated infection can be diagnosed if at least two of the following criteria are present. (1) Significant growth of microorganisms in sonicate or conventional culture of the device. (2) Growth of microorganisms in proximity to the device. (3) Combination of clinical, laboratory, imaging, histopathology, microbiology, and signs of infection^4^. For this study, orthopaedic surgical site infections include incisional and organ/space surgical site infection, and infections of orthopaedic implant such as periprosthetic joint infections, internal fixation associated infections and pin tract infections.

Variables included as risk factors were gender, underlying hypertension, diabetes mellitus, end stage renal failure, anaemia, smoking, types of orthopaedic surgery, duration of surgery and prolonged antibiotic prophylaxis.

The medical records of all the cases and controls were reviewed for risk factors, demographics data and information of the types of operative procedure performed using standard data collection tools. The prevalence of orthopaedic SSI was determined by calculating the percentage of cases of microbiologically proven orthopaedic SSI out of total number of patients who had undergone clean and clean-contaminated orthopaedic surgery during the specified period. The causative organisms isolated from clinical specimen taken intra-operatively and their antimicrobial susceptibility patterns (expressed as percentage resistance) were also determined by reviewing and analysing laboratory data using the in-house laboratory information system (LIS).

The risk factors and outcome of the cases and controls are analysed by chi-square or Fisher’s exact test. The independent risk factors with p value <0.25 were further analysed using a multivariate model. All data analyses were performed using IBM SPSS Statistics Version 24 [International Business Machines Corporation (IBM), Chicago].

## Results

Eighty cases of microbiologically proven orthopaedic surgical site infections were identified from April 2015 to March 2019 in Hospital Universiti Sains Malaysia (HUSM). The prevalence of orthopaedic surgical site infections was calculated using a total of 6436 clean and clean-contaminated orthopaedic operative procedures in 4 years’ duration as denominator, resulting in a local prevalence of 1.243% (1.000% - 1.544%). The most common type of orthopaedics SSI was internal fixation infections, which contribute to 46.25% of the SSIs. Next, superficial SSIs and Prosthetic joint infection accounted for 25.0% and 18.75% of the infections, respectively. Deep SSIs and pin tract infections contribute to 6.25% and 3.75% of the infections, respectively. Overall, SSIs involving lower limbs are more common (70%), compared to SSIs involving the upper limbs (23.75%) and spine (6.25%). For PJIs, the infection of knee (75%) is more common than the infection of hip (25%).

These cases are matched 1:2 with 160 controls. Demographic clinical variables and factors pertaining to the operative procedure were included for risk factor analysis. The risk factors were analysed using univariate logistic regression and their related odds ratios (ORs) and 95% Confidence interval are shown in Table I. Using multivariate model, the risk factors found to be significantly associated with orthopaedic SSI were obesity (OR 3.841, 95% CI 1.668.88) and smoking (OR 6.374, 95% CI 3.617 - 12.839). These are shown in Table II.

**Table I:** Risks factors and outcome in patients with orthopaedic SSI by simple logistic regression

Characteristic / risk factors	Infected (n=80)	Not infected (n=159) **	Crude odds ratio (95% CI)	p value (By fisher exact test)	p value (By simple logistics regression)
Gender					
Male	57 (71.25%)	110 (69.12%)	1.014 (0.612-1.991)	0.742	0.742
Female	23	49			
Hypertension	16 (20.0%)	27 (16.98%)	1.222 (0.615-2.429)	0.567	0.567
Not hypertensive	64	132			
ESRF	2 (2.5%)	5 (3.14%)	0.790 (0.150- 4.163)	0.781	0.781
Not ESRF	78	154			
Prolonged prophylactic antibiotics	70* (89.74%)	135 (84.91%)	1.56 (0.664- 3.642)	0.309	0.309
No prolonged prophylactic antibiotics	8	24			
Clean-contaminated surgery	15(18.75%)	18 (11.32%)	1.808 (0.858-3.810	0.163	0.163
Clean	65	141			
Obesity	20 (25.0%)	13 (8. 02%)	3.744 (1.750-8.006)	<0.001	<0.001
Non-obese	60	146			
Diabetic mellitus	15 (18.7%)	19 (13.57%)	1.70 (0.813 - 3.557)	0.155	0.155
Non- diabetic	65	140			
Anaemia	42 (52.5%)	49 (30.82%)	5.406 (2.839 -10.293)	<0.001	<0.001
Not anaemic	38	110			
Smoking	35 (43.75%)	20 (12.58%)	2.481 (1.427-4.314)	<0.001	<0.001
Non-smoker	45	139			
Prolonged duration of surgery ≥2 hours)	19(23.75%)	67 (42.14%)	2.338 (1.279-4.275)	0.006	0.006
Duration of surgery (<2 hours)	61	92			
Prolonged post-operative stays (≥7days)	45(56.25%)	41(25.79%)	3.700 (2.099-6.523)	<0.001	<0.001
Post-operative stays <7 days	35	118			

**Table II:** Risks factors and outcome in patients with orthopaedic SSI by multiple logistic regression.

Variable	Regression coefficient	Adjusted odds ratio (95% CI)	Wald statistic	p value
Obesity	1.346	3.841 (1.662- 8.88)	9.907	0.002
Smoking	1.852	6.374 (3.167 – 12.839)	26.938	<0.001

Multiple logistic regression model was applied to risk factors that have p value by simple logistic regression of < 0.25

Majority of the cases (86.5%) received prolonged prophylactic antibiotics for a mean duration of 8.48 days (SD 4.94) before surgical site infections were diagnosed, whereas in the control group the mean duration of prophylactic antibiotic was 7.05 days (SD 5.36). However, there was no statistical difference between groups. The most common perioperative prophylaxis regimens used were cefuroxime (81.9%), a combination of cefuroxime and metronidazole (4.7%) and ampicillin-sulbactam (3.8%).

There were 80 isolates from the infected patients. The most common causative agent was Staphylococcus aureus which accounts for 31.25% of the infections. This is followed by Pseudomonas aeruginosa and Enterobacter spp which contribute to 27.5% and 7.5% of the SSIs, respectively. Coagulase negative Staphylococci (7.5%) and Streptococcus pyogenes (5%) are the second and third most common Gram-positive organisms, respectively. Klebsiella pneumoniae which accounted for 6.5% of the cases is the third most common Gram-negative organism. Anaerobic organisms such as Bacteroides fragilis, Finegoldia magna and Clostridium clostridioforme collectively accounted for 3.75% of the cases.

**Fig. 1.**
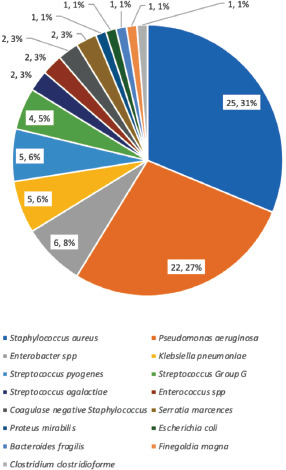
Etiological agents of orthopaedic SSI.

**Fig. 2.**
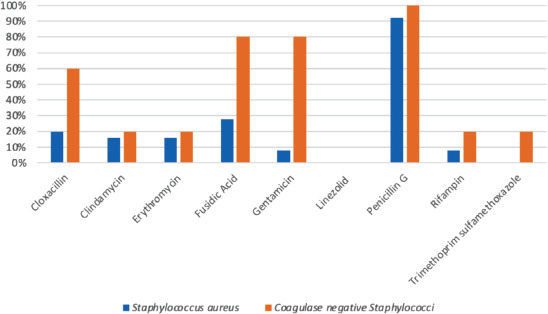
Resistance patterns of most common Gram-positive bacteria causing orthopaedics SSI.

The antimicrobial susceptibility patterns of the most common organisms are summarised in Fig. 1 and 2. Methicillin-resistant Staphylococcus aureus (MRSA) accounted for 20% of the Staphylococcus aureus infections. Majority (60%) of the coagulase negative Staphylococci (CNS) are methicillin resistant. All Streptococcus pyogenes isolates were found susceptible to penicillin, erythromycin, and ceftriaxone. Multidrug resistant (MDR) organisms accounted for 19.4% of the Gram-negative agents, including those that produce extended spectrum beta-lactamases (ESBL) and carbapenems.

*The susceptibility of *Streptococcus pyogenes* against cloxacillin, clindamycin, fusidic acid, gentamicin, rifampicin and trimethoprim-sulfamethoxazole were not tested. The susceptibility of Staphylococci against ceftriaxone was not tested.

**Fig. 3.**
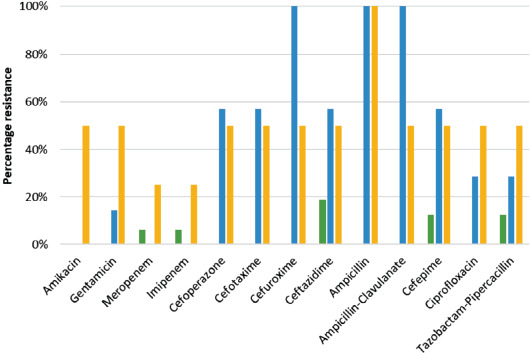
Resistance patterns of the most common Gram-negative bacteria causing orthopaedics SSI.

*Susceptibility of *Pseudomonas aeruginosa* against cefoperazone, cefotaxime, cefuroxime, ampicillin and ampicillin-clavulanate were not tested

## Discussion

Orthopaedic surgery is a surgical field with a diverse type of operative procedures and involve different anatomic locations and the risk of infection may also be affected by the mechanisms of injuries, the underlying pathology and the indications of surgical management.

The exact burden of surgical site infection is not known. However, the Centre of Disease Control (CDC) estimate that the rate of infection is 689.9 cases per 10000 persons (6.89%)^18^. Information regarding the prevalence of orthopaedic surgical site infection is limited. A retrospective study in a tertiary university hospital in Saudi Arabia reported a prevalence of orthopaedic SSI of 2.55%^19^. This study included all orthopaedics procedures including clean and clean-contaminated operations, but excluded SSIs occurring after 30 days, thereby excluding some of the implant associated infections. A teaching hospital in Hong Kong reported a prevalence of 2 - 4.5% in clean and clean-contaminated procedures6. The prevalence of periprosthetic joint infection is estimated to be 0.3 - 1.6%^20^. Internal fixation of closed fracture has the risk of becoming infected in 0.4 - 3.6%^21^ of the cases. The prevalence of wound infection following spinal surgery was found to be 4.2%, with 2.67% being deep surgical site infection^22^. The local prevalence of total orthopaedic SSI is determined by our study to be 1.243%. This is consistent with most of the studies stated above. The higher incidence in some of the studies may be due to the inclusion of contaminated and dirty operative procedures. However, Dhillon *et al* in 1995 reported a clean wound infection rate in a Malaysia teaching hospital of 6.8% with 3.3% of the elective procedures complicated with deep wound infection^23^. A prospective preliminary study in 2018 in a Malaysian tertiary hospital determined that clean and clean-contaminated wound infection rates are 13.9% and 22.0%, respectively^24^. This may represent variations in the local practice and operating environment, temporal difference and differing definitions being used.

The risk factors of surgical site infection have been documented in other studies^25^. Factors associated with SSIs may be host related (such as comorbidities, tobacco use, nutritional status and immunocompetence) or procedure related (such as improper pre-operative skin preparation, inadequate perioperative antibiotic prophylaxis, and degree of trauma to the tissue). The occurrence of SSIs may also be affected by the preventative and infection control measures in place. In addition, orthopaedic implant related infections are uniquely associated to the presence of bacteraemia during the operation, presence of unresolved infections in both adjacent (soft tissue) and distant site (urinary tract) and the degree of contamination and trauma to the skin and soft tissue overlying the surgical site.

In this study, we have identified several risk factors. Using multivariate analysis, factors which are found to be statistically significant are obesity and tobacco use. Obesity is a common and significant risk factor for surgical site infection in general, as well as in orthopaedic practice. This is consistent with the findings in meta-analyses by Yuan *et al*^26^ and Abdallah *et al*^27^ in SSIs in general orthopaedic and spinal surgery, respectively. However, we found that obesity is associated with four times higher risk of SSIs as compared to Yuan *et al*^26^ (two times higher risk). Possible explanation for this is that high body mass index is associated with reduced ambulation post-operatively. This may predispose to wound contamination leading to SSIs. High body mass index (BMI) may also be associated with poor wound healing. Smoking is also an important established risk factors associated with surgical site infection. In orthopaedic practice, smoking is associated with various post-operative complications^28^, both prosthesis-related^29^ and infection-related. In a prospective study by Durand and colleagues, smoking is found to be significantly associated with implant-related orthopaedic surgery^30^. This may be due to the adverse effect of nicotine on bone and wound healing, inhibiting the process of tissue repair. There is evidence that smoking leads to reduced oxygenation and attenuation of wound healing response, leading to diminished bacterial killing^31^.

Anaemia, duration of operation and prolonged postoperative stay was found to be significantly associated with orthopaedic SSIs in univariate analysis but was not a significant risk factor in multivariable analysis. This is consistent with the findings of Voila *et al* that pre-operative anaemia is associated significantly with all post-operative complications and cardiovascular complications in total hip arthroplasty but the association with increased rate of infection is not statistically significant^32^. However, Greenky and colleagues found that pre-operative anaemia is associated with subsequent development of periprosthetic joint infections in a retrospective study of patients who underwent total joint arthroplasty^33^.

Majority of the patients were given a prolonged course of prophylactic antibiotic. We noted that this was a frequent practice in our centre, which was not in accordance with our local guidelines. This practice appeared to be widespread in Malaysia and other countries^34-36^. Such prescribing practice may be due to misconception on the role of prophylactic antibiotic, failure to follow the latest guidelines, peer influence, reliance on habit and personal preferences rather than evidence-based practices and lack of enforcement of institutional policy^36^. The prolonged used of prophylactic antibiotic was found not to be advantageous in preventing SSIs and may be associated with the acquisition of resistant organisms^37^. However, the choice of prophylactic antibiotic in most patients was appropriate as local and international guidelines recommend a first- or second-generation cephalosporin for the prevention of SSIs^38,39^.

Gram positive organisms account for a vast majority of the causative organisms of surgical site infections. This represents the inoculation of surgical site by skin flora. The predominance of Gram-positive organisms as the causative agent of orthopaedic implant related infections may be due to the adherence and biofilm formation on the surface of implanted device by these organisms, leading to persistence of the infection unless the implant is removed. This is consistent with our study which revealed that the most common causative organism in orthopaedic surgical site infection to be Staphylococcus aureus. Not surprisingly, Methicillin-resistant Staphylococcus aureus only accounted for 20% of the Staphylococcus aureus isolated. This probably reflected the proportion of Staphylococci in the hospital that are methicillin-resistant, and by extension the prevalence of MRSA infection in the region^40^.

However, there is a trend of orthopaedic surgical site infections increasingly being caused by Gram negative organisms, as reported by several studies worldwide^41-45^. We found that the most common Gram-negative causative organisms are Pseudomonas aeruginosa and Enterobacter spp. Pseudomonas aeruginosa and most of the Enterobacter species (i.e., Enterobacter cloacae and Enterobacter koseri) are inherently resistant to first- and second-generation cephalosporin. The prolonged use to cefuroxime as perioperative prophylaxis, a controversial practice, may exert selective pressure and lead to the acquisition of antibiotic resistance and the emergence of these Gram-negative organisms as the cause of surgical site infections^46^.

It is likely that orthopaedic SSIs are not entirely preventable as some of the risk factors are not modifiable. However, the incidence of orthopaedic SSIs can be reduced by addressing the modifiable risk factors. Good surgical practices (such as strict adherence to aseptic techniques and adequate skin preparation) and infection control measures (such as appropriate use of prophylactic antibiotic and screening for resistant organisms in high-risk operations) are designed to manage these risk factors, thereby reducing the occurrence of orthopaedic SSIs by eliminating the potentially preventable infections. Surveillance of orthopaedic SSIs is a crucial component of infection control program. By monitoring the rates of procedure related infection, lapses in surgical practices and infection control measures may be detected early. Surveillance may also detect the changing epidemiology and antimicrobial resistance pattern. This may allow for the tailoring and optimisation of prophylactic antibiotic regime at the local institution level for the prevention of orthopaedic SSIs. In addition, educational and awareness programs may be introduced to encourage good surgical and infection control practices in accordance with local and international guidelines.

The study is limited by the retrospective nature and the inclusion of only a single centre, which may restrict the generalisability to the entire country or worldwide. Only microbiologically proven cases are included in this study. The culture negative cases diagnosed by clinical and radiological criteria are not included. However, the associations and trends found in this study may reflect the effect of changing epidemiology or clinical practice on orthopaedic SSI in the country. Therefore, there is a need to conduct a multicentre and prospective study to provide a more representative data and objective evidence to address these issues.

## Conclusion

In conclusion, the prevalence of orthopaedic SSIs in North-eastern Peninsular Malaysia was consistent with other studies done worldwide. We found that tobacco use, and obesity were associated with the occurrence of orthopaedic SSIs. These modifiable risk factors, if actively sought for and addressed prior to operative procedure, may lead to a reduction in the rate of SSI. Although gram positive organisms such as S.aureus accounted for a vast majority of the causative organisms, we found that there was a higher rate of gram negative infections caused by organisms resistant to the prophylactic antibiotics being used. This may be due to the prolonged use of perioperative antibiotic prophylaxis, which exerted selective pressure and led to the acquisition of resistant organisms. A large multicentre prospective study may be needed to address the epidemiology, risk factors and microbiological profile of orthopaedic SSIs in Malaysia more definitively.
